# Hip transposition procedure due to osteosarcoma metastasis of the ilium in a patient with preexisting rotationplasty leads to satisfactory functional result: A case report

**DOI:** 10.1016/j.ijscr.2020.11.072

**Published:** 2020-11-19

**Authors:** Jan Puetzler, Niklas Deventer, Georg Gosheger, Thomas Goesling, Winfried Winkelmann, Tymoteusz Budny

**Affiliations:** a Department of General Orthopedics and Tumor Orthopedics, University Hospital Muenster, Muenster, Germany; b Department of Trauma- and Orthopaedic Surgery, Städtisches Klinikum Braunschweig, Germany

**Keywords:** Osteosarcoma, Hip transposition procedure, Rotationplasty, Bone tumor, Reconstruction

## Abstract

**Introduction:**

The hip transposition is a limb salvage procedure for periacetabular malignancies. Here we present the case of a patient that already had a preexisting ipsilateral rotationplasty.

**Presentation of case:**

A 16 year old male patient with an Osteosarcoma of the left distal femur was treated with wide surgical resection of the tumor and rotationplasty (Salzer/Winkelmann type A1). Despite adjuvant chemotherapy (EURAMOS protocol) he was diagnosed with metachronous metastases in the lung and in the left ilium affecting the acetabulum. As a limb salvage procedure the patient received an internal hemipelvectomy Enneking PI-II and an attachment of the femoral head with a Trevira tube to the sacrum using suture anchors. After six weeks a prosthesis was fitted and gradually full weight bearing was allowed. The patient achieved a good functional result as he was able to walk freely for three more years before he passed away.

**Discussion:**

The hip transposition procedure does not require the implantation of a large tumor prosthesis and thus avoids the problem of an increased risk of infection. Even with preexisting ipsilateral rotationplasty a good functional outcome that allowed the patient full weight bearing could be achieved.

**Conclusion:**

If there are already existing deformities, such as rotationplasty on the same leg, the hip transposition procedure can be considered for periacetabular malignancies, as it can achieve satisfactory results.

## Highlights

• Hemipelvectomy (PI-II) after rotationplasty on the same leg resulted in a good functional result in a 16 year old male patient with osteosarcoma.
• For hemipelvectomy, the *musculus gluteus maximus* is essential for soft tissue coverage, therefore supplying structures must be preserved.
• The triradiate cartilage of the acetabulum closes around a bone age of 12 years in boys and then is no sufficient barrier for an osteosarcoma.

## 1 Introduction

The hip transposition is a limb salvage procedure for periacetabular malignancies that leads to satisfactory functional results and does not require the implantation of large tumor prostheses that are associated with an increased risk of infection. However, this procedure was never described in a patient that already had Borggreve-Van Nes Rotationplasty on the same leg.

The work was reported in line with the SCARE 2018 criteria [1].

## 2 Presentation of case

A 16 year old boy without previous diseases and drug history, non-smoker and without genetic predisposition presented with load dependent pain and swelling of his left thigh. An extra articular Osteosarcoma of the left distal femur was diagnosed after radiological imaging (MRI scan, radiography) and incisional biopsy. He wished to receive a biological reconstruction via rotationplasty after speaking to other patients that reported satisfaction with this method. After a wide surgical resection of the tumor, reconstruction of the left leg was therefore achieved with a Borggreve-Van Nes Rotationplasty performed by the leading surgeon Gosheger, classified by Salzer and later Winkelmann as type A1 [2], [3]. This procedure comprised plate-fixation of the 180° externally rotated lower leg to the femur shaft to allow the ankle to function as a knee joint. The medial and lateral heads of the gastrocnemius were augmented to the origins of the rectus femoris and sartorius muscles at their origins. The patient received adjuvant chemotherapy according to the EURAMOS protocol to lower the risk for tumor recurrence (pre-operatively two 5-week cycles of cisplatin 120 mg/m^2^, doxorubicin 75 mg/m^2^, methotrexate 12 g/m^2^ × 2 (MAP) and post-operatively two further cycles of MAP and two cycles of MA). The femoro-tibial fusion healed uneventful and the patient started full weight bearing with an exoprosthesis.

**Fig. 1:**
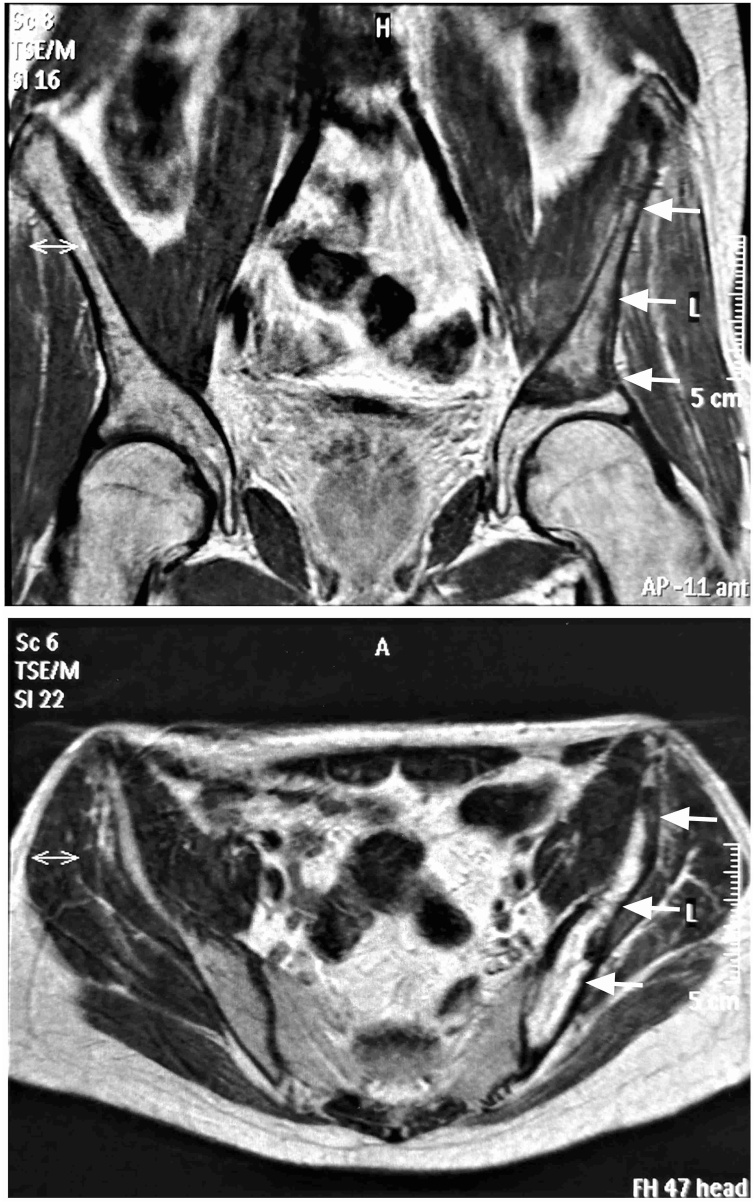
MRI of the pelvis, extension of the osteosarcoma metastasis in the left ilium (arrows) in coronal (top) and axial (bottom) view before internal hemipelvectomy.

**Fig. 2:**
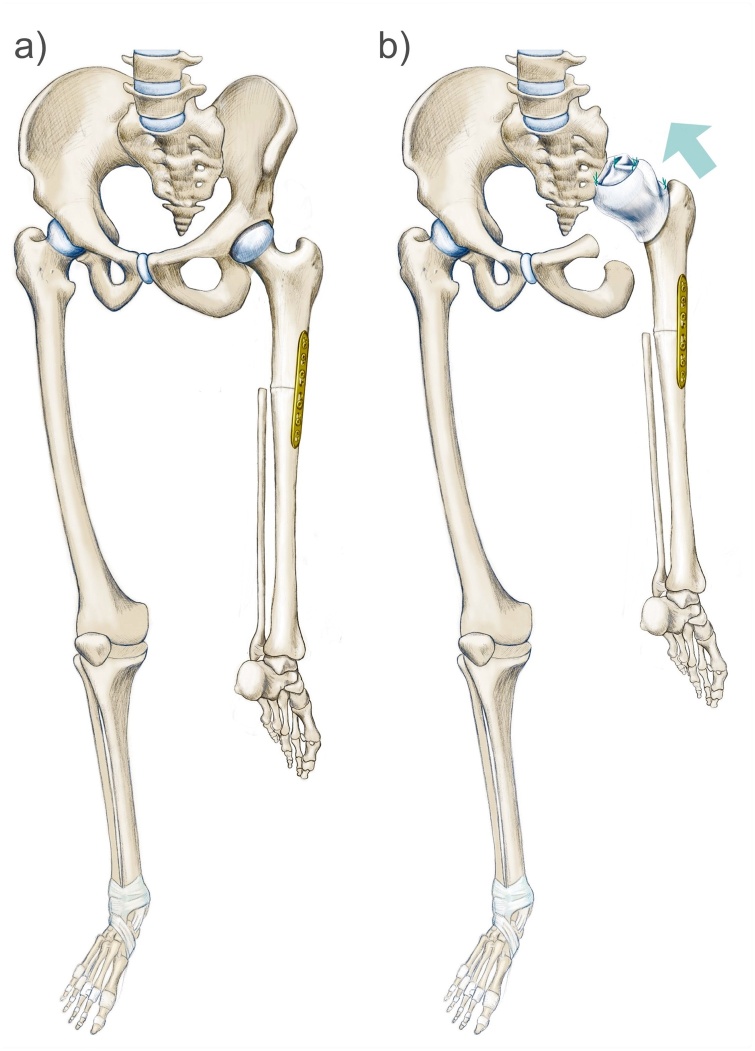
Schematic drawing of the preexisting Borggreve-Van Nes rotationplasty (left) and the hemipelvectomy PI-II and hip transposition type 2a (right), attaching the femoral head wrapped into a Treviva tube (polyethylene terephthalate mesh) and suture anchors to the sacrum.

**Fig. 3:**
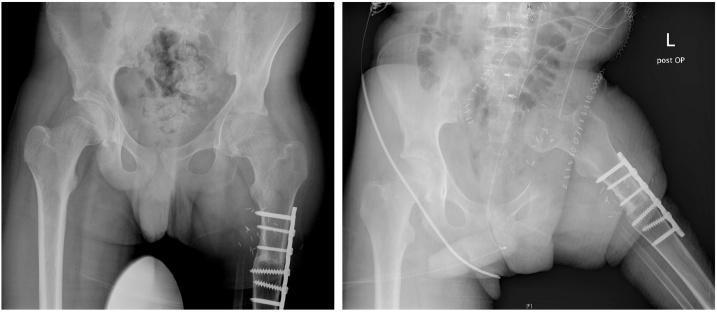
Radiographs of the pelvis in a.p. direction before (left) and after (right) hemipelvectomy PI-II and hip transposition procedure type 2a.

One year later the patient presented with fatigue and pain in the left pelvis. He was diagnosed with a recurrence of the osteosarcoma presenting with lung metastasis and a huge metastasis of the left Ilium extending close to the acetabulum (Fig. 1). These metastases were not visible on the initial MRI one year earlier. Due to the young patient age and a high demand for a good functional result, the decision was made to resect the iliac metastasis via internal hemipelvectomy type I-II according to Enneking (including the ilium and the acetabulum), leaving on the left side of the pelvis the sacrum and partially in pubic bone and ischium [4]. The supra iliac crest approach from the sacrum to the groin was used by Gosheger as described by Winkelmann et al. [5]. Reconstruction was performed via the hip transposition procedure (Type IIa) forming an artificial capsule with a Trevira tube (polyethylene terephthalate mesh, Implantcast, Buxtehude, Germany) that attaches the proximal femur with cortical bone anchors to the sacrum [6]. The femoral head was wrapped directly into the mesh and fixed with tight non-absorbable sutures as described earlier (Fig. 2, Fig. 3) [7]. A type 1 hip transposition procedure, where remaining parts of the acetabulum are attached to the sacrum to allow better containment of the transposed femoral head, was not possible, as, meaning this structure was no sufficient barrier for the osteosarcoma anymore.

The *musculus gluteus maximus* is essential for soft tissue coverage, therefore supplying structures were preserved. A further inevitable shortening of the leg of 4 cm occurred, however due to the massive shortening that the patient had already coped with after the rotationplasty, this new condition was relatively quickly tolerated by the patient.

**Fig. 4:**
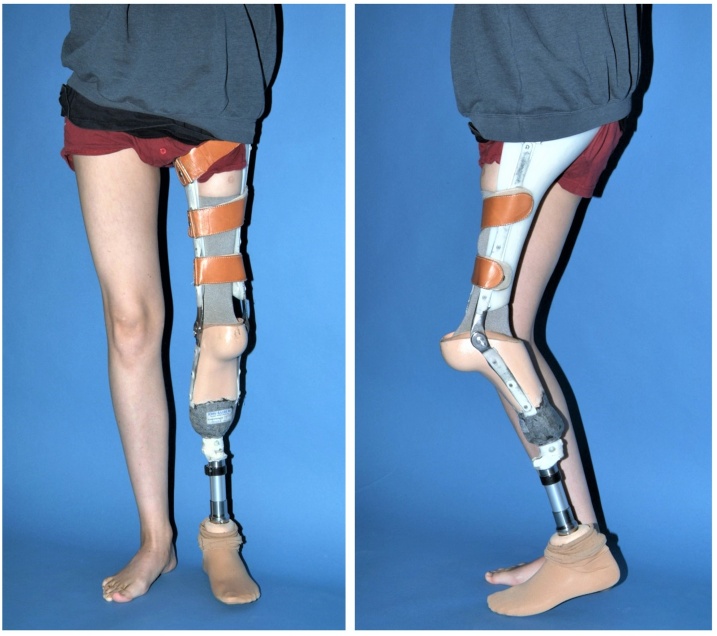
Patient standing with prosthesis after hip transposition procedure.

After one wound revision due to delayed wound healing at the cranial portion of the approach, the wound healed uneventful. Scar tissue formed in the wound bed and grew into the Trevira tube, thus stabilizing the neo-hip joint. After six weeks of resting in bed without a cast, the patient gradually started to mobilize. A custom-made prosthesis was fitted, and the patient started to walk with crutches with increasing weight bearing within the pain free margins. He tolerated the postoperative phase very well. After another two months the patient was walking freely at home and used crutches only for his comfort when leaving home (Fig. 4). His walking distance with the prosthesis was unlimited and he was he was satisfied with the outcome. However, three years later he died after a rapid disease progress despite additional chemotherapy. He kept the ability to walk with prosthesis until one week before passing away. Written informed consent was obtained from the patient’s mother for publication of this case report and accompanying images. A copy of the written consent is available for review by the Editor-in-Chief of this journal on request.

## 3 Discussion

The hip transposition procedure is an established treatment option for periacetabular tumors of the pelvis as it allows a biological and functional reconstruction of pelvic defects after wide resection [4], [5].

In this case the patient had a recurrence of the osteosarcoma in the ilium and pulmonary metastases resulting in a very poor life expectancy. The 5-year overall survival in the case of lung metastases is approximately 30%, compared to 70% in patients without metastasis [8], [9], [10].

A procedure was needed that allowed early rehabilitation, good function and also a relatively low risk for major complications, primarily infection [11]. The hip transposition procedure in general achieves good functional results especially in young patients compared to no reconstruction (flail hip) and at the same time poses a lower risk for infection compared with endoprosthetic reconstruction [11].

Superificial skin infection or delayed wound healing seem to be the norm rather than the exception due to the extensive approach and soft tissue and bone resection [12]. Therefore, adequate dead space management and soft tissue coverage is essential to prevent deep infection. The *musculus gluteus maximus* was used to cover the Trevira tube. Skin closure was fascilitated by the inevitable shortening of the leg of 4 cm.

In general, to achieve a functionally good result after internal hemipelvectomy the preservation of the femoral nerve, arteria and vein is necessary. In this case with preexisting rotationplasty the ankle joint served as a knee joint, thus the protection of the sciatic nerve was very important to achieve movement of the exoprosthesis. The preservation of the sciatic nerve also prevented from development of phantom pain compared to external hemipelvectomy.

## 4 Conclusion

This is the first description of a hip transposition procedure as a limb salvage procedure in a patient that had rotationplasty on the same leg one year earlier, resulting in a good function and unlimited walking distance for three more years before the patient passed away. Thus, the hip transposition procedure can be considered in cases of periacetabular malignancies, despite preexisting leg deformities such as an ipsilateral rotationplasty.

## Declaration of Competing Interest

The authors report no declarations of interest.

## Funding

The authors did not receive any additional funding for this publication.

## Ethical approval

This case report is exemt from ethical approval in our institution.

## Consent

Written informed consent was obtained from the patients mother (legal guardian) for publication of this case report and accompanying images. A copy of the written consent is available for review by the Editor-in-Chief of this journal on request. Please see consent section in instructions to authors for further information.

## Registration of research studies

Not applicable for this case report.

## Guarantor

Gosheger Georg, Puetzler Jan.

## Provenance and peer review

Not commissioned, externally peer-reviewed.

## CRediT authorship contribution statement

**Jan Puetzler:** Conceptualization, Writing - original draft. **Niklas Deventer:** Investigation, Supervision. **Georg Gosheger:** Investigation, Project administration, Supervision. **Thomas Goesling:** Investigation. **Winfried Winkelmann:** Writing - review & editing. **Tymoteusz Budny:** Writing - review & editing.

## References

[1] Agha R.A. (2018). The SCARE 2018 statement: updating consensus Surgical CAse REport (SCARE) guidelines. Int. J. Surg..

[2] Winkelmann W.W. (1996). Rotationplasty. Orthop. Clin. N. Am..

[3] Salzer M. (1981). Treatment of osteosarcomata of the distal femur by rotation-plasty. Arch. Orthop. Trauma Surg..

[4] Enneking W.F., Dunham W.K. (1978). Resection and reconstruction for primary neoplasms involving the innominate bone. J. Bone Jt. Surg. Am..

[5] Winkelmann W. (1988). Eine neue Operationsmethode bei malignen Tumoren des Darmbeins. Z. Orthop. Unfall..

[6] Gebert C., Gosheger G., Winkelmann W. (2009). Hip transposition as a universal surgical procedure for periacetabular tumors of the pelvis. J. Surg. Oncol..

[7] Gebert C. (2011). Hip transposition as a limb salvage procedure following the resection of periacetabular tumors. J. Surg. Oncol..

[8] Huang X. (2019). Risk and clinicopathological features of osteosarcoma metastasis to the lung: a population-based study. J. Bone Oncol..

[9] Briccoli A. (2010). High grade osteosarcoma of the extremities metastatic to the lung: long-term results in 323 patients treated combining surgery and chemotherapy, 1985–2005. Surg. Oncol..

[10] Huang Y.-M. (2009). The metastasectomy and timing of pulmonary metastases on the outcome of osteosarcoma patients. Clin. Med. Oncol..

[11] Puchner S.E. (2017). Oncological and surgical outcome after treatment of pelvic sarcomas. PLoS One.

[12] Angelini A. (2014). Infection after surgical resection for pelvic bone tumors: an analysis of 270 patients from one institution. Clin. Orthop. Relat. Res..

